# The master's apprentices- making best practice out of practically nothing!

**DOI:** 10.1186/2050-2974-1-S1-P11

**Published:** 2013-11-14

**Authors:** Mandy Yiu, Ruth Willis, Maggie Phillips

**Affiliations:** 1 The Paediatric Unit, Flinders Medical Centre, Australia

## 

The Paediatric Unit at Flinders Medical Centre in South Australia caters for children aged between 0 - 18 years of age, within a large general metropolitan hospital. Consistent with national trends we have experienced a significant increase in adolescents admitted with an eating disorder. In response a core eating disorder group visited other hospitals within the Room: State and interRoom: State in the hope of gaining insight into how to best manage this cohort. We formed a management team consisting of Paediatric Consultant, Psychiatrist, Nursing and Allied Health, CAHMS, School Teacher and Diversional Therapist. With much trepidation, February 18th 2013 saw the launch of our Eating Disorder program, heavily based on the Westmead Children's Hospital model with their on-going guidance and support. Our immediate focus has been on delivering education sessions for all staff; developing written resources for staff, patients and families. Imperative to the success of this program has been setting up a weekly activity program, family meetings, team management meetings and addressing ward management issues.

This presentation will describe our journey and specific challenges we met along the way to ensure we are meeting not only the needs of these complex patients but also empowering staff and families to deal with concerns on a daily basis.

